# Low-grade serous carcinoma metastasizing to the chest wall and incidental Müllerian inclusion in a retrosternal lymph node 16 years after the initial ovarian serous borderline tumor: a case report

**DOI:** 10.1016/j.gore.2026.102160

**Published:** 2026-07-03

**Authors:** Yu Miyama, Haruka Omori, Kousuke Uranishi, Masataka Hirasaki, Tomonori Kawasaki, Akira Yabuno, Tomoaki Torigoe, Hiroyuki Nitanda, Yoshinao Kikuchi, Yuko Sasajima, Masanori Yasuda, Taku Homma

**Affiliations:** a Department of Pathology, Saitama Medical University International Medical Center, Japan; b Division of Biomedical Sciences, Research Center for Genomic Medicine, Saitama Medical University, Japan; c Department of Clinical Cancer Genomics, International Medical Center, Saitama Medical University, Japan; d Department of Gynecological Oncology, Saitama Medical University International Medical Center, Japan; e Department of Orthopaedic Oncology & Surgery, Saitama Medical University International Medical Center, Japan; f Department of General Thoracic Surgery, Saitama Medical University International Medical Center, Japan; g Department of Pathology, Teikyo University School of Medicine, Tokyo, Japan; h Department of Clinical Pathology, Teikyo University School of Medicine, Tokyo, Japan

**Keywords:** Serous borderline tumor, Low-grade serous carcinoma, Nodal Müllerian inclusion, Endosalpingiosis, chest wall, sternum, rib

## Abstract

**Background:**

Ovarian serous borderline tumors (SBTs) generally exhibit indolent behavior; however, a small subset of SBTs progresses to low-grade serous carcinoma (LGSC), in some cases after prolonged follow-up. Notably, very late progression with distant metastasis has rarely been reported.

**Case presentation:**

A 50-year-old woman with a history of bilateral ovarian SBTs presented with a chest wall mass 16 years after initial diagnosis. At 34 years of age, the initial left ovarian tumor was treated with fertility-sparing surgery and pathologically diagnosed as an SBT pT1c3 because of intraoperative capsule rupture and positive ascitic cytology. After 5 years, the contralateral side lesion was resected, and the patient was diagnosed with recurrent SBT. Sixteen years after the initial diagnosis, imaging revealed a calcified mass at the right sixth costochondral junction. Surgical resection revealed papillary to micropapillary carcinoma with destructive invasion consistent with metastatic LGSC. The tumor cells were positive for estrogen receptor, PAX8, and WT-1, with wild-type p53 expression. A nearby lymph node showed micro-metastasis consisting of a few tumor cells. In addition, an incidentally resected retrosternal lymph node was identified as a nodal Müllerian inclusion. Recurrent SBT, LGSC, and a nodal inclusion commonly harbored *KRAS* hotspot mutations.

**Conclusion:**

This case highlights the potential for the very late malignant progression of ovarian SBTs and the need for long-term surveillance. In addition, the unique location of a nodal inclusion and its clonal relationship with LGSC provide further insights into the pathogenesis of ectopic Müllerian tissues.

## Highlights

• A small subset of SBTs may progress to LGSC after a prolonged interval.
• A rare case of chest wall LGSC occurring 16 years after ovarian SBT is described.
• Nodal Müllerian inclusion had the same *KRAS* hotspot mutation as SBT and LGSC.
• Potential of relationship between Müllerian inclusion and serous tumor development.

## Graphical abstract

**Figure:**
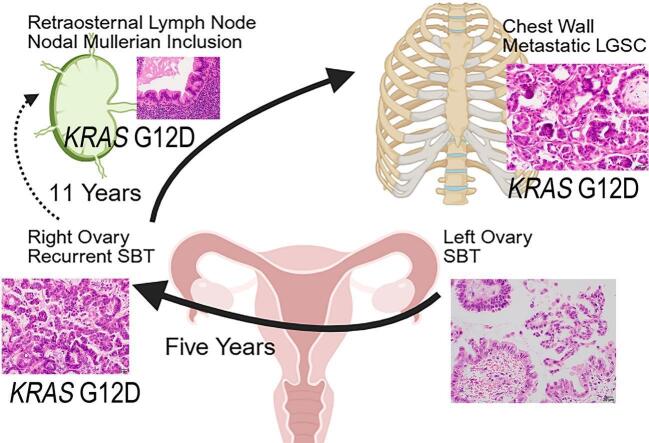

## 1 Introduction

Serous borderline tumors (SBTs) of the ovary represent a distinct category of serous neoplasms, characterized by epithelial proliferation and nuclear atypia without destructive stromal invasion (WHO Classification of Tumours Editorial Board, 2020). Although the overall prognosis is favorable, approximately 2–5% of cases eventually progress to low-grade serous carcinoma (LGSC), often after a prolonged clinical course (Longacre et al., 2005). The identification of clinicopathological risk factors for disease progression remains challenging. We report a rare case of bilateral ovarian SBTs that progressed to LGSC metastasizing to the chest wall 16 years after the initial diagnosis. Notably, nodal Müllerian inclusion was incidentally identified in a nearby retrosternal lymph node. This case highlights the indolent yet progressive nature of SBTs and indicates a potential molecular relationship between serous tumors and nodal lesions.

## 2 Case presentation

**Fig. 1:**
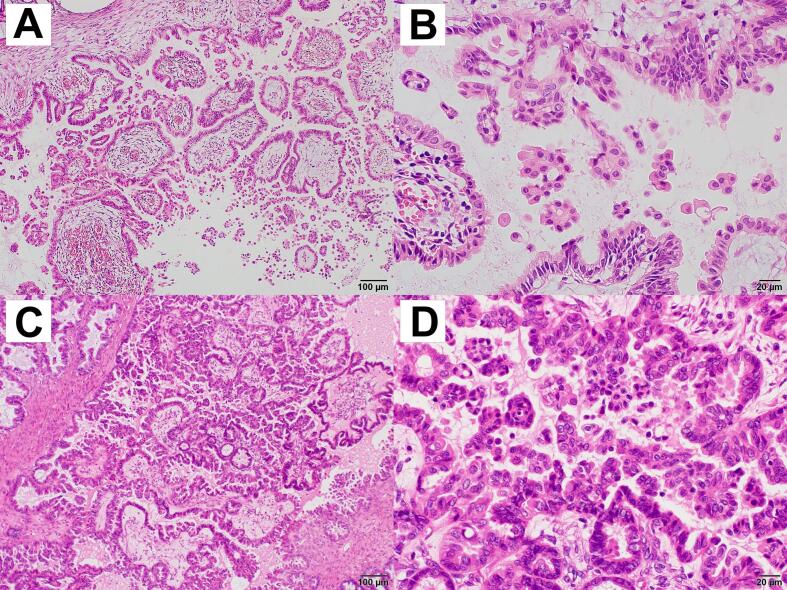
Microphotographs of initial and recurrent ovarian serous borderline tumors. (A, B) Left ovarian serous borderline tumor at 34 years of age. The tumor formed stratified papillae (A). Tumor cells are mildly atypical with fine, slightly increased chromatin. (C, D) Recurrent right ovarian SBT at 39 years of age. The recurrent tumor forms stratified and complex papillae (C). The cellularity is higher, and the chromatin is coarser than that of the initial tumor. Severe nuclear atypia is not observed.

A 50-year-old woman had a significant medical history of an ovarian tumor. At 34 years of age, she underwent fertility-sparing surgery for a left ovarian tumor. Histological examination revealed an SBT with papillary architecture, mild nuclear atypia, focal micropapillary growth, and no stromal invasion (Fig. 1A and B). The tumor was staged as pT1c3 based on intraoperative capsule rupture and positive ascitic cytology.

At 39 years of age, a contralateral right ovarian mass was detected on pelvic magnetic resonance imaging (MRI), showing a mixed solid and cystic tumor measuring approximately 67 × 54 mm, accompanied by a small amount of ascites. The patient underwent a total hysterectomy and right salpingo-oophorectomy. The recurrent tumor formed complex papillae (Fig. 1C). In the high-power view, the cellularity was higher, and the chromatin was coarser than that of the initial tumor (Fig. 1D). A focal micropapillary architecture measuring less than 5 mm was identified at several sites. The tumor was diagnosed as a recurrent SBT (pT1c3) based on positive ascites cytology.

**Fig. 2:**
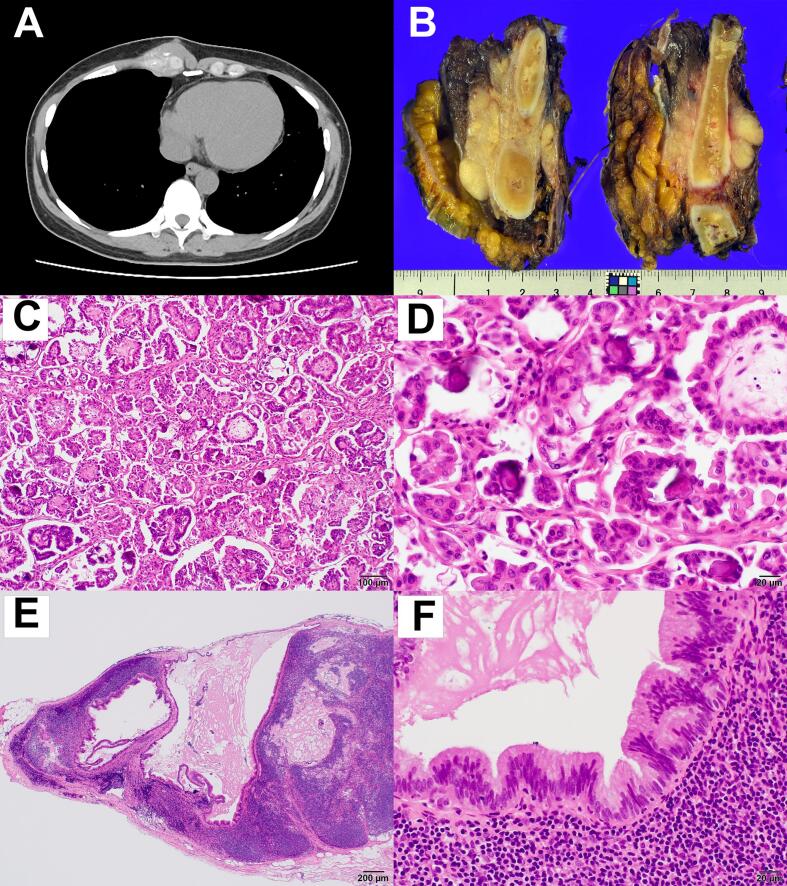
Radiographic, macroscopic, and microscopic findings of metastatic low-grade serous carcinoma of the chest wall and microphotographs of nodal Müllerian inclusion. (A) Chest computed tomography (CT) showing a 4.5-cm soft-tissue mass with calcification at the right sixth costochondral junction extending to and invading the subcutaneous tissue. (B) Gross appearance of the resected chest wall specimen demonstrating a 4.5-cm yellow-white tumor spanning the rib. (C) Histological findings showing papillary to micropapillary tumor growth with slit-like spaces and destructive infiltration into the surrounding tissue. (D) Tumor cells exhibit enlarged nuclei with loss of polarity and mildly increased chromatin; psammoma bodies are also present. (E) Incidentally detected and resected retrosternal lymph nodes. Dilated glandular structures containing mucinous material are observed within each node. (F) The glands are lined by tall columnar epithelial cells with cilia and mucin. Nuclear atypia is mild, and nuclear polarity is well preserved. The lesion shows an endocervical gland-like morphology consistent with nodal Müllerian inclusion.

The patient was followed up on an outpatient basis. Ten years after the second surgery, the serum CA125 levels gradually increased, although no definite lesions were identified on routine imaging. Sixteen years after the initial diagnosis, positron emission tomography-computed tomography and MRI revealed a 4.5-cm mass with calcification at the right sixth costochondral junction (Fig. 2A). The chest wall tumor was surgically resected.

## 3 Pathological findings

Gross examination of the resected chest wall specimen revealed a 4.5-cm yellow-white lobulated mass extending across the rib (Fig. 2B). Histologically, the tumor showed papillary-to-micropapillary growth with slit-like spaces and destructive invasion (Fig. 2C) involving the skeletal muscle. The tumor cells exhibited enlarged nuclei with a loss of polarity and mildly increased chromatin. Psammoma bodies were also observed (Fig. 2D). These features are consistent with those of LGSCs.

A small regional lymph node showed micrometastatic LGSC composed of fewer than 10 tumor cells (Supplementary Fig. 1). Immunohistochemical studies revealed diffuse positivity for estrogen receptor (ER), PAX8, and WT-1, with a wild-type p53 staining pattern, supporting Müllerian differentiation (Table 1).

**Table 1:** Comparison of results between initial, recurrent, and metastatic tumors and benign Müllerian inclusions.

Age		34	39	50	
Surgery		LSO	RSO + TH	Local excision	Excision
Location		Left ovary	Right ovary	Chest wall	Retrosternal lymph node
Pathological diagnosis		SBT	Recurrent SBT	Metastatic LGSC	Müllerian inclusion
Immunohistochemistry	PAX8	N/A	N/A	diffuse+	diffuse+
	CK7	N/A	N/A	diffuse+	diffuse+
	CK20	N/A	N/A	negative	negative
	ER	diffuse+	diffuse+	diffuse+	diffuse+
	WT-1	diffuse+	diffuse+	focal+	focal+
	Ki-67	5%	5%	10%	3%
	p53	N/A	N/A	wild-type pattern	wild-type pattern
	BRAF V600E	N/A	N/A	wild-type pattern	N/A
	MTAP	N/A	N/A	retained	N/A
Molecular	*KRAS* G12D (Exon 2 c.35G > A)	N/A	Present (NGS)	Present (NGS)	Present (Sanger)

ER, estrogen receptor; LGSC, low-grade serous carcinoma; LSO, left salpingo-oophorectomy; N/A, not assessed; NGS, next-generation sequencing; RSO, right salpingo-oophorectomy; SBT, serous borderline tumor; TH, total hysterectomy.

In addition, an incidentally resected retrosternal lymph node demonstrated dilated glandular structures lined by ciliated and mucinous columnar epithelia with minimal nuclear atypia, resembling cervical glandular epithelia (Fig. 2E and F). Combined with the immunohistochemical results, the nodal lesion was interpreted as an ectopic Müllerian inclusion.

## 4 Molecular findings

We determined the genetic differences between recurrent SBT of the right ovary and LGSC of the chest wall through whole exome sequencing using formalin-fixed paraffin-embedded samples. Exome enrichment utilized the Twist Exome 2.0 Kit (Twist Bioscience HQ, South San Francisco, CA, USA), and the prepared DNA libraries were sequenced using NovaSeq 6000 (Illumina, San Diego, CA, USA) with paired-end 151 bp reads at Azenta Ltd. (Tokyo, Japan).

Both contained the *KRAS* hotspot mutation located in exon 2, c.35G > A (G12D) (Supplementary Table 1). No other pathogenic mutations were identified. Furthermore, we detected the same *KRAS* G12D mutation in the nodal Müllerian inclusion of the retrosternal lymph node by DNA extraction using laser microdissection and Sanger sequencing (Supplementary Fig. 2). Table 1 presents the pathological and molecular results for the series of lesions.

## 5 Discussion

We encountered a case of SBT progressing to LGSC in the chest wall 16 years after the initial diagnosis of ovarian tumor. To the best of our knowledge, this is the first case of LGSC metastasizing to the chest wall. Likewise, only a single case of cardiophrenic lymph node metastasis, instead of chest wall involvement, has been reported (Ragusa et al., 2011). According to Longacre et al., long-term follow-up of 276 patients identified advanced FIGO stage, microinvasion in the primary tumor, invasive implants, and a micropapillary/cribriform pattern as risk factors for recurrence (Longacre et al., 2005). Prat et al. further reported that micropapillary architecture alone was not an independent risk factor; rather, its association with invasive implants was predictive of progression (Prat and De Nictolis, 2002). Among patients with stage I SBT who underwent conservative treatment, younger age (<30 years), tumor bilaterality, and cystectomy have been associated with an increased risk of recurrence (Uzan et al., 2014). As demonstrated in this case, prolonged follow-up should be considered, particularly in young patients who undergo fertility-preserving surgery.

Although the common *KRAS* mutation was observed in the recurrent SBT and LGSC in this case, no additional pathogenic variants were identified at the progression of LGSC. This finding supports the concept of clonal continuity and indicates that malignant transformation in at least a subset of cases may occur without the acquisition of further driver mutations (Jones et al., 2012). Consistent with this notion, previous studies have proposed that progression from SBT to LGSC may be driven by alternative molecular mechanisms, including copy number alterations, loss of 9p21 involving the *CDKN2A/2B l*ocus, epigenetic dysregulation, and changes in tumor–stromal interactions or epithelial–mesenchymal transition–related pathways (Emmanuel et al., 2014; Wong et al., 2012; Shih Ie and Kurman, 2004). SBT is divided into two molecular subtypes: carcinoma-like and benign-like, indicating that a subset of SBTs initially has malignant potential (Curry et al., 2014). Collectively, these observations indicate that genetic progression in low-grade serous neoplasia is not necessarily mutation-driven and may instead reflect cumulative non-mutational molecular events that facilitate invasion and tumor progression.

A Müllerian inclusion in the retrosternal lymph node was incidentally detected. Nodal Müllerian inclusions, such as endosalpingiosis or endocervicosis, are often observed in SBT, with 30–33% frequency (Messini et al., 2019; Djordjevic et al., 2010). In addition, nodal inclusion harbored the *KRAS* mutation commonly observed in both recurrent SBT and LGSC. This phenomenon has been reported by Chui et al., with nodal endosalpingiosis accompanied synchronously with SBT/LGSC harboring common *KRAS/BRAF* mutations in 91% (11/12) cases, indicating the potential of nodal endosalpingiosis for neoplastic transformation upon bypass of endogenous oncosuppressive mechanisms (Chui and Shih, 2020). In this context, two main hypotheses have been proposed to explain the pathogenesis: metaplasia and migration theories. The former indicates that Müllerian metaplasia of ectopic tissues, such as mesothelium, occurs independently of the associated ovarian tumor. This mechanism could also account for the presence of shared mutations in synchronous SBT/LGSC, as ectopic tissues may be susceptible to similar hormonal and genetic influences as their native counterparts (Wetterwald et al., 2023).

However, this explanation may not fully account for our case because of the distinct biological environment of the pleura compared with that of the pelvis. Notably, nodal salpingiosis is predominantly identified in retroperitoneal lymph nodes, including para-aortic (34%), external iliac (12%), and common iliac (9%) regions (Reich et al., 2000), whereas solitary Müllerian inclusions within the thoracic lymphatic system are exceedingly rare. Although retroperitoneal lymphadenectomy was not performed and the status of retroperitoneal lymph nodes remains uncertain, we favor the migration hypothesis. Specifically, Müllerian inclusions with shared *KRAS* mutations may have disseminated through lymphatic pathways and subsequently involved the retrosternal lymph node, reflecting a process analogous to the lymphatic spread observed in SBT/LGSC. However, nodal inclusions are generally regarded as non-neoplastic lesions, as they are typically detected incidentally and are not associated with adverse clinical outcomes (Burla et al., 2022).

There is a limitation in establishing clonality among SBT, LGSC, and nodal Müllerian inclusions. Given the high prevalence of *KRAS* mutations in both SBT and LGSC, the presence of an identical *KRAS* mutation alone does not provide definitive evidence of a direct lineage relationship.

## 6 Conclusion

In conclusion, we report a case of long-standing ovarian SBT that progressed to LGSC. Fertility-sparing surgery, capsular rupture, and positive ascites were considered potential risk factors for recurrence or progression. Although the recurrent SBT demonstrated increased architectural complexity and cellularity compared to the primary tumor, a micropapillary or cribriform pattern was not prominent in either lesion. Incidental nodal inclusions in a poststernal lymph node harbored the same *KRAS* mutation as the recurrent SBT and LGSC. These unusual locations and shared clonal features may support the hypothesis of migratory potential of ectopic Müllerian tissue, which can retain a benign phenotype over a prolonged period.

## CRediT authorship contribution statement

**Yu Miyama:** Writing – original draft, Project administration, Data curation, Conceptualization. **Haruka Omori:** Visualization. **Kousuke Uranishi:** Software, Formal analysis. **Masataka Hirasaki:** Software, Formal analysis. **Tomonori Kawasaki:** Investigation. **Akira Yabuno:** Visualization. **Tomoaki Torigoe:** Resources. **Hiroyuki Nitanda:** Resources. **Yoshinao Kikuchi:** Resources. **Yuko Sasajima:** Validation. **Masanori Yasuda:** Writing – review & editing. **Taku Homma:** Supervision.

## Ethics statement

Written informed consent was obtained from the patient for the publication of this case report and the accompanying images.

## Funding

This study was supported by the departmental research fund.

## Declaration of competing interest

The authors declare that they have no known competing financial interests or personal relationships that could have appeared to influence the work reported in this paper.

## Data Availability

All data are available including supplementary information.
