# Supplementary material for: Low-grade serous carcinoma metastasizing to the chest wall and incidental Müllerian inclusion in a retrosternal lymph node 16 years after the initial ovarian serous borderline tumor: a case report
Source: Gynecol Oncol Rep. 2026 Jul 3;66:102160. doi: 10.1016/j.gore.2026.102160 (PMC13380180; doi:10.1016/j.gore.2026.102160)
Supplement: Supplementary file 1 — Supplementary material [file mmc1.docx]

Supplementary Figure 1. Micrometastasis of low-grade serous carcinoma.

A small regional lymph node with a 1.2-mm diameter lesion contains a cluster of tumor cells (arrowhead) (A). Tumor cells floating in a lymphovascular vessel appear to be a micrometastasis (B).


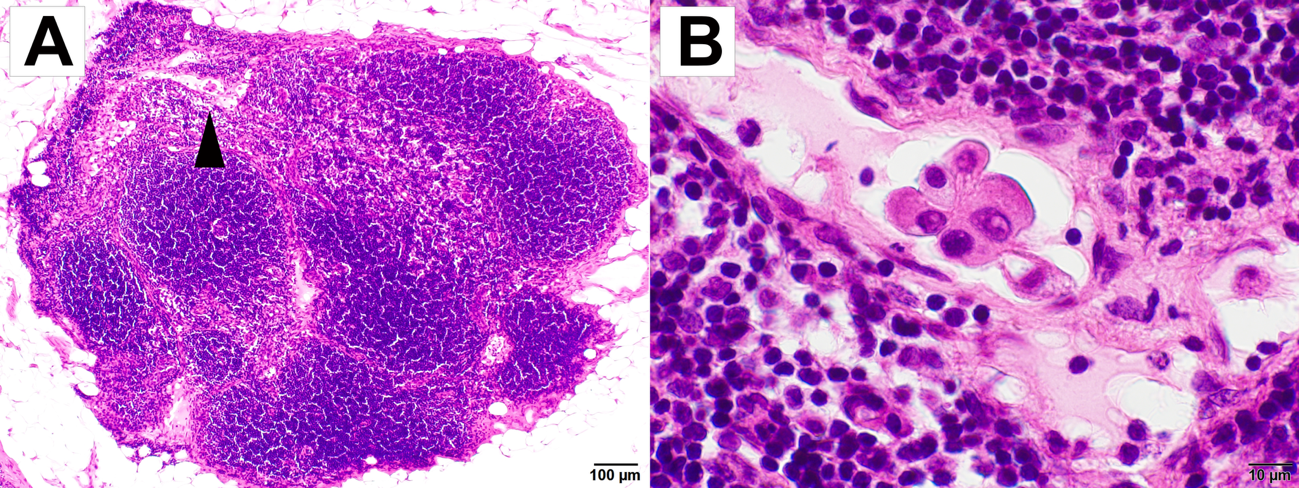


**Supplementary Figure 2. Chromatogram of *KARS* exon 2 in Müllerianosis of the retrosternal lymph node.**

Sanger sequencing analysis was performed using DNA extracted through laser microdissection. A heterozygous hotspot mutation in KRAS exon 2 (c.34G>A; p.G12D) was identified.


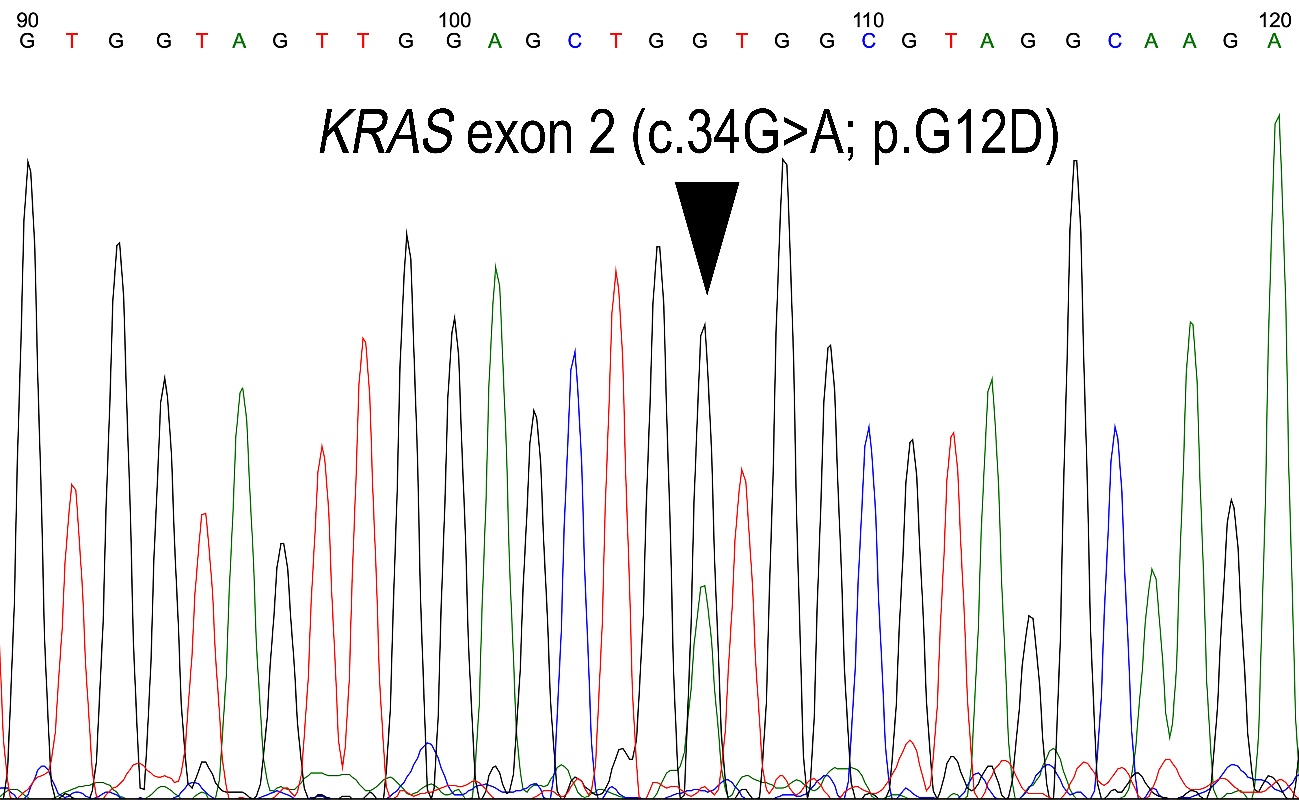


Supplementary Table 1. Exonic functional gene variants in recurrent serous borderline tumor (SBT) and metastatic low-grade serous carcinoma (LGSC) using whole-exome sequencing

|  |  |  |  | **Recurrent SBT** | | |  | **Metastatic LGSC** | | |
| --- | --- | --- | --- | --- | --- | --- | --- | --- | --- | --- |
| Gene symbol | ExonicFunc.refGene | Nucleotide Change | AA change | RefSeq count | AltSeq count | VAF (%) |  | RefSeq count | AltSeq count | VAF (%) |
| ***KRAS*** | nonsynonymous SNV | NM_001369786:exon2:c.35G>A | p.G12D | 20 | 16 | 44 |  | 53 | 33 | 39 |

AA, amino acid; SNV, single nucleotide variant; VAF, variant allele frequency
